# Play Mode Effect of Exergames on Subthreshold Depression Older Adults: A Randomized Pilot Trial

**DOI:** 10.3389/fpsyg.2020.552416

**Published:** 2020-10-26

**Authors:** Jinhui Li, Yin-Leng Theng, Schubert Foo

**Affiliations:** ^1^School of Journalism and Communication, Jinan University, Guangzhou, China; ^2^National Media Experimental Teaching Demonstration Center, Jinan University, Guangzhou, China; ^3^Wee Kim Wee School of Communication and Information, College of Humanities, Arts, and Social Sciences, Nanyang Technological University, Singapore, Singapore

**Keywords:** social interaction, elderly, exercise games, Wii, mental health

## Abstract

**Background:**

Subthreshold depression is a common mental disorder in late life. Increasing studies have supported the positive effects of exergames to subthreshold depression. The current study aims to investigate how play mode potentially affects exergames’ effects on subthreshold depression among older adults.

**Method:**

A between-group experiment was carried out to compare the effect of exergames with different play modes. Fifty-two Singaporean older adults with subthreshold depression were randomly assigned into two conditions, and performed either single-player or multiple-player Nintendo Wii Tennis exergames for 6 weeks, while the key variables of depression, social support and loneliness were measured at both pre- and post-study period.

**Results:**

Findings from path analysis suggested that older adults in multiple-player exergames experienced lower levels of loneliness, and further more reduction on subthreshold depression, when compared to those in single-player exergames. Although social support was not affected by play mode, the significant relationship among social support, loneliness, and depression was found in the context of exergaming.

**Conclusion:**

This study not only provides additional insight into a possible causal association lining play mode and health outcomes of exergames, but also opens the discussion of how to optimize antidepressive effect of exergames for older adults.

## Introduction

Subthreshold depression, or minor depression, is generally defined as a cluster of depressive symptoms, in which the number, duration, or quality is insufficient to meet the DSM-IV criteria of major depression (Bali and Jiloha, 2008). Although being below the clinical criteria of major depression, subthreshold depression often has a much higher prevalent rate across all age groups, particularly in aging population (Meeks et al., 2011). Subthreshold depression leads to significant negative outcomes in late life, including poorer physical health and even increased mortality (Montross et al., 2008).

With the advent of interactive digital technology, exergames, which combine the digital gaming and physical exercising (Oh and Yang, 2010), has become a popular alternative to traditional exercise programs and now are increasingly used in healthcare domains (Gil-Gomez et al., 2011). Recent research has examined the positive impacts of exergames on psychosocial well-beings among older adults, including improvements in loneliness and mood (Wollersheim et al., 2010; Kahlbaugh et al., 2011), self-esteem, and positive affect (Jung et al., 2009). In a systematic review, Li et al. (2015) have confirmed that exergames have medium effect on reducing the symptoms of depression among older generations. Increasing studies (Rosenberg et al., 2010; Zhou et al., 2012) have extended the positive effects of exergames to subthreshold depression. Another study from Li et al. (2017) further implied that exergames led to greater reduction on subthreshold depression among older adults, when compared to traditional exercise. However, the lack of depth of study in this area calls for further research that explores the influential factors that may affect their anti-depressive effects.

Several game studies examined the effects of exergames factors on attitudes, performance and physical conditions (O’Donovan et al., 2012; Park et al., 2014). However, to our knowledge, there is no particular study investigating the factors that influence the exergames’ impacts on depression. The current study is a pilot study with the aim to contribute to this new research domain, by investigating how one key factor in exergames – play mode – potentially affects its effect on subthreshold depression among older adults.

Play mode is an important theme in game studies. Play mode is usually categorized in video games history into two types: single-player mode (SP) and multiple-player mode (MP). SP refers to a particular game mode designed to be played by a single player, whereas MP refers to that designed to be played by two or more players simultaneously. Many previous studies investigated the effects of play mode on players’ motivation, performance and in-game experience (Smyth, 2007; Peng and Hsieh, 2012; Chen et al., 2015). Player modes were also supported to affect a player’s psychosocial attributes in real life, such as social support (de Kort and Ijsselsteijn, 2008; Uz and Cagiltay, 2015) and loneliness (Kahlbaugh et al., 2011). Social support and loneliness are common depression predictors supported by previous literature (Nezlek et al., 1994; Ingram et al., 1999; Singh and Misra, 2009). Hence, play mode in exergames may affect depression through the mediation effects of social support and loneliness. The below section provides the theoretical perspective for the assumptions of play mode effect on depression and related psychosocial attributes.

## Literature Review and Hypotheses

### Social Support, Loneliness, and Depression

In social gerontology literature, the link between social support and depression is well established. *Buffering Hypothesis* (Cohen and Wills, 1985) is the most influential theoretical perspective on social support, which hypothesizes that social support reduces the effects of stressful life event on health through either the supportive actions of others or the belief that support is available (Lakey and Cohen, 2000). In other words, the existence of one’s social network, as well as substantive interactions generated among social ties, can buffer people from negative and stressful events. Therefore, social support acts to reduce the chances of stressors and negative events that provoke depression.

A sizable number of aging studies have established the relationship between social support and geriatric depression (Verstraten et al., 2005; Lee et al., 2012; Su et al., 2012). Given that a person’s social network decreases over time in late life (Antonucci, 1991), older adults receive less emotional support from social networking. This could increase the risk of depression. Barg et al. (2006) found that high adequacy of social support that older adults receive corresponds to low depressive scores. In a study with forty elderly aged 60 years and above, Patil et al. (2014) found a significant negative correlation between perceived social support and depression.

Besides the direct effect on geriatric depression, social support would also influence geriatric depression through loneliness. Loneliness refers to the subjective feeling state of being alone, separated, or apart from others (Tomaka et al., 2006). It is one of the strongest predictors of depression according to Singh and Misra (2009). Strong association between loneliness and geriatric depression was further confirmed in a large scale study (Aylaz et al., 2012). The longitudinal study of Cacioppo et al. (2006) supported that loneliness is a significant risk factor for depression among the aging population. Cohen-Mansfield and Parpura-Gill (2007) proposed a framework, *Model of Depression and Loneliness*, to examine factors influencing loneliness and depressed affect in older population. According to this framework, the reduced social contacts that occurs with age will influence loneliness, which, in turn, will affect depression. The feeling of loneliness is common for elderly people in their late life because of the lack of close family ties (e.g., living alone), impaired social support, and loss of mobility in social activities. The negative effect of social support on loneliness among older adults has found in many studies, especially on the Asian contexts (Kang et al., 2018; Chen et al., 2019). Lonely people suffer from more depressive symptoms, as they have been reported to be less happy, less satisfied, and more pessimistic (Singh and Kiran, 2013). As a result, loneliness may play a mediation role on the relationship between social support and depression among older adults.

### Role of Play Mode

In video games, social support is likely to be affected by different play mode. Many researchers have examined the social support derived from video games with multiple players (Trepte et al., 2012; Zhang and Kaufman, 2016a). In contrast with those in single-player games, players in multiple-player games need to learn and apply their social skills to achieve game goals. While interacting with others, players enhance their in-game social networking and interaction in these virtual social communities. This phenomenon is more obvious in games designed specifically for engaging cooperation and mutual assistance (Ewoldsen et al., 2012). In these collaborative games, players in a team assist each other and share successes and failures together. These social interactions strengthen group cohesion and interdependent bonds among teammates (Isbister, 2010; Banks, 2012), and foster strong feelings of virtual support and new friendships (Smyth, 2007). These virtual support and relationships in multiple-players gaming may affect and extend players’ pre-existing relationships in real life (Williams, 2006). Gentile and Gentile (2008) argued that social skills learned and practiced in the gaming environment could be generalized into the real context, which may promote prosocial behavior and enhance social support in daily life. Trepte et al. (2012) has presented a large-scale study to examine the theoretical framework of how social interactions in online multiple-player gaming affected offline social support. In their study, players’ social proximity and mutual familiarity during online interactions were supported to foster both online bridging social capital (people feel informed and inspired by each other) and bonding social capital (emotional support and understanding), while both social capital dimensions are positively related to offline social support.

The theoretical framework of gaming and social support provided above can be applied into the context of exergaming and older adults. It is reasonable to posit that exergames in multiple-player mode lead to higher level of social support than those in single-player mode, and the higher level of social support further results in greater reduction of depression among older adults.

Similar to social support, play mode in video games may affect loneliness through social interaction and communication among players. Li and Counts (2007) argued that MP games could actually increase communication between teammates via digital and traditional channels. Further, such game mode could create feelings of co-presence and make players feel more connected to each other (Lazzaro, 2004). The co-presence and connection to others could therefore reduce loneliness. A systematic review from Li et al. (2018) identified that playing MP exergames could improve older adults’ social well-being, through increasing social bonding with their peers and grandchildren. In the study from Kahlbaugh et al. (2011), older residents received personal visits from students who played a bowling game on Nintendo Wii console with them for 10 weeks. Results showed that older residents felt less lonely than they had at the start of the study. The decrease in loneliness was perhaps not due to playing the Wii itself, but by the interactions between older participants and students. In a large sample survey study, Lee and Ishii-Kuntz (1987) indicated that doing something together with other people reduced loneliness among older adults.

As a result, compared to SP exergames, the social interactions and communication in MP exergames lead to lower level of loneliness, and thus results in greater reduction of depression among older adults.

### Purposes of the Study

**FIGURE 1:**
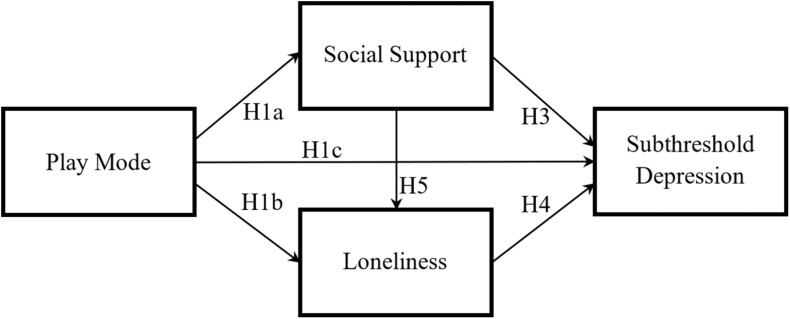
Proposed conceptual model.

This study sought to build a conceptual model to address the influencing mechanism between exergame play mode and depression in old age. The model is rooted in cognitive-behavioral theories and conceptualizes depression as resulting from an interaction of social events and cognitive processes. It utilizes two key concepts, social support and loneliness, to clarify how play mode can facilitate the depression treatment in the exergaming context. Figure 1 shows the proposed conceptual model in this study. Based on the model, five hypotheses were proposed in the specific context of exergames and subthreshold depression in late life:

H1: Compared to SP exergames, MP exergames lead to (a) a higher level of social support; (b) a lower level of loneliness; and (c) a lower level of subthreshold depression among older adults.

H3: Social support is negatively associated with subthreshold depression among older adults.

H4: Loneliness is positively associated with subthreshold depression among older adults.

H5: Social support is negatively associated with loneliness among older adults.

H6: Both (a) Social support and (b) loneliness mediate the effect of play mode on subthreshold depression among older adults.

## Materials and Methods

### Participants

Participants were recruited from two senior activity centers located in the western and northern parts of Singapore, respectively. The center managers assisted in delivering recruitment message and approached potential participants. Generally, older adults were included in the study if they were aged 55 and above, and diagnosed with subthreshold depression in the screening section. Patient Health Questionnaire – 9 (PHQ-9; Spitzer et al., 1994) was used as a screening tool for admission into the study. Followed the scale instructions from Kroenke and Spitzer (2002), the total PHQ-9 score below “4” indicated none depression, whereas “5” to “14” indicates mild to moderate depression, and “15” to “27” indicates moderately severe to severe depression. Therefore, only participants who scored 5–14 in PHQ-9 were included in the final pool. Given that the particular interventions, participants were asked to self-report their previous experience on exergaming system. Only those with no experience or little experience (less than a few hours performance) were included in the study. Further, through self-reporting and information obtained from center managers, participants were excluded if they had the following conditions which prevent them from performing the exergame correctly and safely: (a) have serious cognitive disorders (e.g., Parkinson disease or Alzheimer’s disease), (b) intellectual disability and physical limitations (severe mobile, visual, or hearing problems), (c) other depressive disorders (e.g., bipolar, dysthymic, schizophrenia), or (d) have received antidepressant medications (or other forms of depression therapies) within the last 3 months.

Based on a systematic review of Li et al. (2015) which examined the overall effect size of exergames on depression, a general effect size of 0.25 was used in the current study to determine the sample size. Through the G^∗^Power software with *a priori* analysis method, a sample of 54 participants is required in order to achieve the according general effect size with a power of 0.9 (α = 0.05). What’s more, the results of this systematic review shown that majority of previous studies used a small sample size of less than 50 participants. As a result, the current study targeted on 60 participants (30 per condition) which is slightly higher than recommended number.

In randomization process, each involved participant was labeled with a unique ID after screening, and an online tool^1^ was used to generate two experimental conditions with random participant IDs. In order to avoid the interaction, these two experimental conditions in the same center were conducted in different time slots.

The ethical approval of this study was obtained from the Institutional Review Board at the university where the research team was originally from (IRB-2014-07-039). Participation in this pilot study was voluntary, and informed consent was obtained for all participants before the study. Each participant was awarded SGD$15 equivalent of shopping voucher after having completed all sessions.

### Interventions

To manipulate play mode, two different exergame conditions – SP and MP – were involved in the study. Nintendo Wii Tennis was chosen as the exergame for both conditions because of two reasons. First, according to previous literatures (Gao and Mandryk, 2011; Theng et al., 2012), older adults were recommended to perform physical activities with simple and single movements because of their impaired physical and cognitive conditions. The Wii Tennis game fulfils these safety criteria and has already been used as exercise intervention for older adults in previous studies (Rosenberg et al., 2010; Maillot et al., 2012). Second, the tennis game contains both SP and MP modes, which could ensure equal comparison between two conditions.

In SP condition, participants were asked to perform the exergames individually and played against a virtual player. Due to practical considerations on schedule and arrangement, participants came with a group of 15 people in one session and took turns to play the exergames. Despite the group setting, each participant was instructed to play on their own and without any physical assistance from others during the gaming. The total time for a group was around 3 h in one session. In the MP condition, two participants formed a team and cooperated in the same game to play against two virtual players. Similar to SP condition, all participants in came with a group of 14 people (seven pairs of participants) and performed the exergames in one session. To balance the performance time with the SP condition, the total time for MP condition was around 1.5 h for each session. The MP mode in the study applied collaborative games but not competitive ones. Compared to collaborative mode with the shared objective, competitive mode with the personal objective may elicit anxiety and fear of failure (Wu et al., 2015). These emotions elicited in competitive games may have negative effects on depression.

Participants from both conditions performed the corresponding exergame interventions once a week for 6 consecutive weeks. During the intervention period, one or two student assistants coordinated each exergame session. They assisted in setting up the exergame and solved the technical problems occurred during the game playing.

### Outcome Measures

Participants were asked to fill in two self-reported surveys at both pre-study (before the first exergame section) and post-study period (after the last exergame section). The basic demographic information of participants was collected in pre-study survey, including their age, gender, education level, living conditions, and physical health conditions. The three key variables, including subthreshold depression, social support, loneliness, were collected at both pre-study and post-study period. Trained student assistants from the research team assisted participants who had literacy and visual problems in filling the questionnaires. Both English and Chinese versions of questionnaire were used in the study. Most of the measurements were adopted from existing sources to ensure the validity. Participants selected the language version that matched their preference.

#### Subthreshold Depression

The PHQ-9 (Spitzer et al., 1994) is a self-administrated depression scale under the Primary Care Evaluation of Mental Disorders (PRIME-MD), which is a widely used diagnostic screening instrument in primary care. In addition to recognizing major depression, PHQ-9 is also a useful tool for detecting subthreshold depression in the general population (Martin et al., 2006). The validity and reliability as diagnostic and assessing measurements are well-established in previous studies (Kroenke et al., 2001; Löwe et al., 2004). The tool uses a four-point Likert scale (from 0 “Not at all” to 3 “Nearly every day”) to measure items such as “Little interest or pleasure in doing things” or “Feeling down, depressed or hopeless.” The total score ranges from 0 to 27, with a higher score indicating higher severity of depression. The Chinese version of PHQ-9 was adopted directly from the PHQ website^2^ organized by Pfizer Inc. The Cronbach’s α of PHQ in pre- and post-study are 0.57 and 0.70, respectively.

#### Social Support

Social support was measured by the perceived social support subscale from Berlin Social-Support Scales (BSSS; Schulz and Schwarzer, 2003). Good reliability of BSSS was also found among elderly population (Patil et al., 2014). The BSSS subscale comprises eight items assessing emotional and instrumental aspects of social support. Participants indicate their agreement with the statements like “I know some people upon whom I can always rely” on a four-point Likert scale, from 1 “Very strongly disagree” to 7 “Very strongly agree.” The total score ranges from 8 to 56, and a higher add-up score indicates higher level of perceived social support. The Chinese version of BSSS was developed through back translations conducted by three Chinese doctoral students majored in communication studies. The Cronbach’s α of BSSS in pre- and post-study are 0.87 and 0.82, respectively.

#### Loneliness

The University of California Los Angeles (UCLA) Loneliness Scale (Russell, 1996) is commonly used for measuring loneliness of respondents including older adults. The present study applied a short form (8 items) of UCLA Loneliness Scale (ULS-8; Hays and DiMatteo, 1987). ULS-8 consists of eight items selected according to the results of an exploratory factor analysis. The scale has high internal consistency and high correlation with the original scale and other related measures (Hays and DiMatteo, 1987). The ULS-8 was revised to suit the elderly participants in current study. The scale employs a four-point Likert scale with values ranging from 1 “Never” to 4 “Always.” The total score of ULS-8 ranges from 8 to 32. No cut-off score was identified to define loneliness, but a higher score on this scale indicates a more intense feeling of loneliness. The Chinese version of the ULS-8 used in the study was developed from previous works (Chou et al., 2005; Zhou et al., 2012). The Cronbach’s α of ULS-8 in pre- and post-study are 0.64 and 0.73, respectively.

### Statistical Analysis

Descriptive statistics of key variables were first conducted for both pre- and post-study data, following by a series of mixed analysis of variance (mixed ANOVA) to examine the interaction effects between time (pre-study vs. post-study) and play mode (SP game vs. MP game). Time was input as a within-group variable while play mode as a between-group variable. Both the descriptive statistics and mixed ANOVA tests were performed in IBM SPSS version 23.

Path analysis was then applied to test the conceptual model proposed between play mode and subthreshold depression. Path analysis is one of the most common techniques used in structural equation modeling (SEM). SEM is a family of statistical methods with a special aim to develop and test theoretical models. SEM evaluates the relationships between observed and latent variables (Hoyle, 1995). In path analysis, goodness-of-fit is first evaluated, followed by the estimation of path coefficients. Path coefficients, also known as standardized regression coefficients, determine the effects of exogenous (predictor) variables on endogenous (predicted) variables.

Different from common SEM in cross-sectional designs, the conceptual model in the current study involved a categorical exogenous variable (play mode). Bollen (1989) clarified that the inclusion of categorical exogenous variables did not violate the assumption of multivariate normality underlying the commonly used maximum likelihood method of estimation. MacCallum and Austin (2000) further indicated that one can use SEM to model the relationships of experimentally manipulated independent variables (such as dummy variables), to other variables, including covariates, mediators, and outcomes. All path analyses were conducted in Mplus version 6.0 (Muthén and Muthén, 1998).

## Results

**FIGURE 2:**
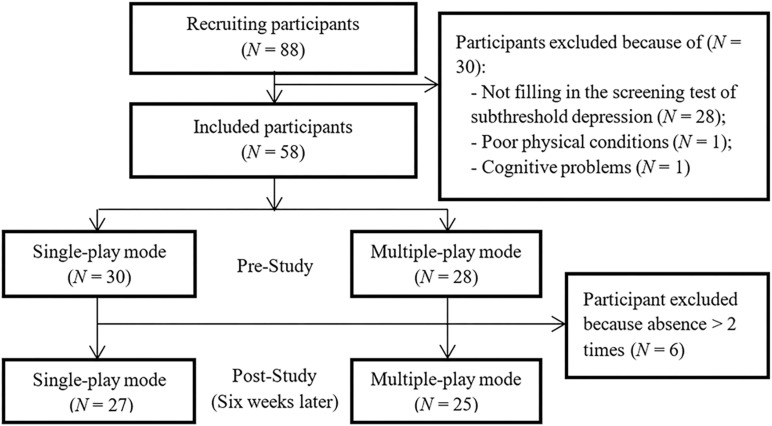
Flow chart of the participants.

Fifty-eight participants met the inclusive criteria and were involved in actual study. During the 6-week intervention period, six participants were reported to be absent in more than two sessions, due to the reasons including loss of interest (two participants), conflict schedule (three participants), or poor health condition (one participant). They were considered as drop-out and did not included in the final analysis. Figure 2 illustrates the flow chart of the participants in the study.

### Descriptive Statistics

A total of 52 participants completed both pre- and post-study surveys. The mean age of included participants was 72.12 (SD = 8.65), and 39 (75%) of them were female participants. Descriptive analysis was conducted across the two conditions on demographic characteristics and key psychosocial variables at pre-study period. Table 1 illustrates the detailed results. Findings from *t*-test and Chi-square tests indicated that participants from the two conditions did not have significant differences in demographic characteristics, as well as physical and cognitive status. Furthermore, no significant group difference emerged during the pre-study period in depression [*t*(50) = 0.56, *p* = 0.582], social support [*t*(50) = 1.24, *p* = 0.221], and loneliness [*t*(50) = 1.62, *p* = 0.111]. As a result, participants in SP and MP can be considered as equal in demographic, physical, and psychosocial conditions. Table 2 indicates key psychological outcomes across two conditions at both pre-study and post-study period. Findings indicates significant bivariate correlations among subthreshold depression, social support and loneliness after the intervention period.

**TABLE 1:** Descriptive analysis across two conditions in study two (*N* = 52).

	**Single-player (*N* = 27)**	**Multiple-player (*N* = 25)**	**Difference across conditions**
**Demographics**			
Age*	70.78 (8.37)	73.56 (8.88)	*t*(50) = −1.16, *p* = 0.250
55–64 years	5 (18.5%)	5 (20.0%)	–
65–74 years	14 (51.9%)	11 (44.0%)	–
75 years and above	8 (29.6%)	9 (36.0%)	*–*
Gender (Female)	20 (74.1%)	19 (76.0%)	χ^2^(1) = 0.03, *p* = 1.000
Education (≤Primary school)	19 (70.4%)	22 (88.0%)	χ^2^(1) = 2.42, *p* = 0.177
Living condition (Alone)	3 (11.1%)	8 (32.0%)	χ^2^(1) = 3.40, *p* = 0.093
Exercise frequency (≤Once per week)	9 (33.3%)	14 (56.0%)	χ^2^(1) = 2.70, *p* = 0.162
**Physical Health**			
Eye sight*	2.37 (0.63)	2.52 (0.77)	*t*(50) = −0.77, *p* = 0.445
Hearing*	2.93 (1.14)	2.92 (0.86)	*t*(50) = 0.02, *p* = 0.983
Mobility status*	3.04 (1.09)	2.40 (0.76)	*t*(50) = 2.42, *p* < 0.05
Cognitive status*	3.11 (1.01)	2.64 (0.64)	*t*(50) = 1.99, *p* = 0.052

*Data presented as Mean (Standard Deviation).

**TABLE 2:** Description of depression, social support, and loneliness (*N* = 52).

	**1**	**2**	**3**	**Cronbach’s alpha α**	**Total**	**Single-player (*N* = 27)**	**Multiple-player (*N* = 25)**
**Pre-study period**							
1. Depression (PHQ-9)	–			0.57	6.63 (2.42)	6.81 (2.35)	6.44 (2.52)
2. Social support (BSSS)	−0.73**	–		0.87	39.48 (7.21)	40.67 (6.53)	38.20 (7.82)
3. Loneliness (ULS-8)	0.26	−0.09	–	0.64	13.31 (3.25)	14.00 (3.66)	12.56 (2.60)
**Post-study period**							
1. Depression (PHQ-9)	–			0.70	2.73 (1.95)	3.22 (1.95)	2.20 (1.85)
2. Social support (BSSS)	−0.41**	–		0.82	44.54 (5.11)	44.07 (5.19)	45.04 (5.09)
3. Loneliness (ULS-8)	0.69**	−0.34*	–	0.73	11.12 (2.49)	11.78 (2.26)	10.40 (2.57)

Data are presented as Mean (Standard Deviation). *p < 0.05, **p < 0.01.

### Mixed ANOVA Results

**FIGURE 3:**
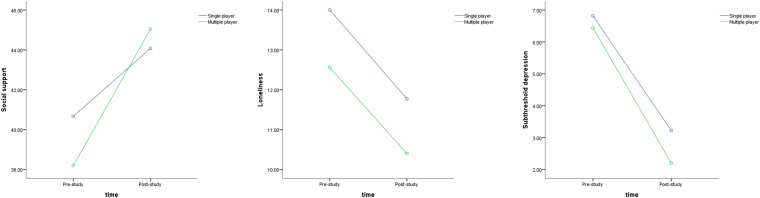
Interaction effects from ANOVA results.

The results of the mixed ANOVA did not show any significant interaction effects between time and play mode on social support [*F*(1,50) = 2.710, *p* = 0.106, η^2^ = 0.051], loneliness [*F*(1,50) = 0.005, *p* = 0.944, η^2^ = 0.00], or subthreshold depression [*F*(1,50) = 0.802, *p* = 0.375, η^2^ = 0.016]. Figure 3 demonstrated the plots of the interaction effect among the three psychological variables. Despite the MP condition seemed to release more increase in social support and more reduction in subthreshold depression over the 6 weeks when compared to SP condition (Inspected from the descriptive statistics in Table 2), these changes were not statistically significant.

Nevertheless, there were strong significant main effects of time on all the three key psychosocial attributes, including social support [*F*(1,50) = 24.152, *p* < 0.001, η^2^ = 0.326], loneliness [*F*(1,50) = 25.027, *p* < 0.001, η^2^ = 0.334], and subthreshold depression [*F*(1,50) = 117.431, *p* < 0.001, η^2^ = 0.701]. The findings indicated that the older adults had improvements on social support, loneliness, and subthreshold depression after the 6-week exergames playing. As a result, the strong effects of exergames were supported as a psychosocial intervention for older adults with subthreshold depression.

### Model Examination

In path analysis, goodness-of-fit was first assessed through multiple fit statistics, including Chi-square test, Comparative Fit Index (CFI), Root Mean Square Error of Approximation (RMSEA), and Standardized Root Mean Square Residual (SRMR). Chi-square test (χ^2^) is the most basic fit statistic that compares predicted covariance matrix with observed matrix (Bentler, 2004). To reduce the effects of sample size, normed Chi-square (χ*^2^/df*) was calculated. A value of normed Chi-square smaller than 3 indicated an acceptable fit. CFI assesses the relative improvement in fit of the researcher’s model compared with a baseline model (Hu and Bentler, 1990). Values greater than roughly 0.90 may indicate reasonably good fit of the proposed model. RMSEA measures error of approximation (Steiger and Lind, 1980) with a value less or equal to 0.05 considered as good fit and 0.05–0.08 considered as fair fit. SRMR is a measure of the mean absolute value of the covariance residuals. A recommended cut-off point for SRMR is smaller than 0.08 (Hu and Bentler, 1990).

**FIGURE 4:**
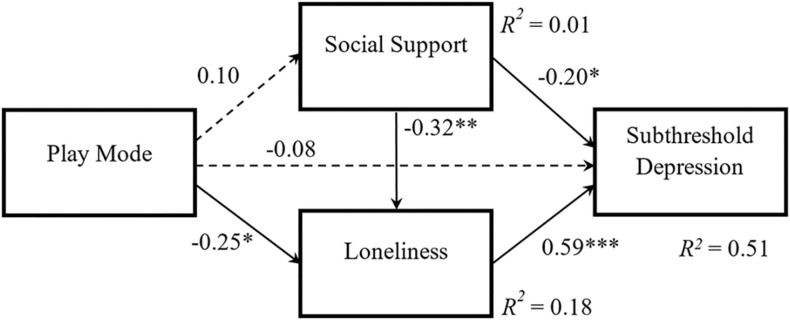
Results of path analysis in the final model. Path coefficients are standardized. The solid line indicates a significant path with ^∗^*p* < 0.05, ^∗∗^*p* < 0.01, ^∗∗∗^*p* < 0.001. The dashed line indicates non-significant path with *p* > 0.05.

Based on the above criteria, the proposal model resulted in a good fit to data. Table 3 shows the goodness-of-fit indices and model fit results. Estimations of path coefficient and hypotheses testing were conducted after determining the final model. Figure 4 shows the results of the path analysis in the final model. During the hypotheses testing, significant negative effects of exergame play mode were observed on loneliness among older adults (β = −0.25, SE = 0.12, *p* = 0.042), thereby supporting H1b. However, H1a was not supported with no significant play mode effects on social support (β = 0.10, SE = 0.14, *p* = 0.488). Consequently, compared to SP exergames, MP exergames led to more reduction in loneliness among older adults, but not in social support. A strong and significant predictive effect from loneliness was also observed on subthreshold depression among older adults (β = 0.59, SE = 0.09, *p* < 0.001). Meanwhile, subthreshold depression was also significantly affected by social support, with β = −0.20, SE = 0.10, *p* = 0.047. Thus, both H3 and H4 were supported. However, the direct effect of exergame play mode was not significant on subthreshold depression among older adults (β = −0.08, SE = 0.10, *p* = 0.434), thereby not supporting H1c. Lastly, H5 was supported by a significant path from social support to loneliness (β = −0.32, SE = 0.12, *p* = 0.008), which highlights the potential effect of social support on loneliness during exergame playing.

**TABLE 3:** Goodness-of-fit indices and model fits.

	**χ^2^**	***df***	***p***	**χ*^2^/df***	**CFI**	**RMSEA**	**SRMR**
Recommended values	N/A	N/A	>0.05	<3.0	>0.9	<0.080	<0.080
Proposed model	0.607	1	0.4359	0.607	1.000	<0.001	0.019

*df, degree of freedom; CFI, comparative fit index; RMSEA, root mean square error of approximation; SRMR, standardized root mean square residual.*

Following the guideline by Stride et al. (2015), the mediation tests were further conducted on Mplus with Andrew Hayes’ PROCESS analysis. The mediation results indicated that play mode in exergames has a (approaching) significant indirect effect on subthreshold depression via medication of loneliness, with β = −0.15, SE = 0.08, *p* = 0.055. But the mediation role of social support was not supported in the relationship between play mode and subthreshold depression, with a non-significant indirect effect of β = −0.02, SE = 0.03, *p* = 0.511. Consequently, the results from the model examination supported H6b but not H6a.

A significant large *R*^2^ of 0.51 (*p* < 0.001) in subthreshold depression was reported during the post-study period. This result demonstrated that 51% of the unique variance of subthreshold depression was explained by independent and mediating variables mentioned above. Generally, the findings from path analysis support the final model in which four out of the six path coefficients are statistically significant. To sum up, compared to SP exergames, MP exergames reduced subthreshold depression via the effect on loneliness. Although social support was not supported to be a significant mediator of play mode, it independently affected loneliness and subthreshold depression among older adults.

## Discussion

The pilot study extended the discussion of play mode from in-game experience to mental health improvements. The study also introduced this important theme into the new domain of exergames for health. First of all, the significant improvements on depression and its related attributes between pre- and post-study period have supported the overall effectiveness of exergames as an emerging depression intervention for older adults. These findings were consistent with previous exergame studies (Rosenberg et al., 2010; Chao et al., 2015). It was well established that the physical activity triggers the release of certain body chemicals (such as β-endorphins or dopamine), which consequently result in the improvement of mood and feeling of well-being (Delgado and Moreno, 2000; Brosse et al., 2002).

More importantly, the study investigated the possible effect of play mode (SP vs. MP) on subthreshold depression among older adults through a between-group controlled study. The research serves as a pioneer study to examine the factors that mediate anti-depressive effects of play mode in exergames. Overall, the proposed mediation model in the study was generally supported by the path analysis. Good model fit and high *R*^2^ of dependent variable further confirmed the robustness of the final model. In this model, play mode was revealed to have indirect effects on subthreshold depression among older adults, which was mediated by loneliness. Accordingly, older adults who performed MP exergames experienced lower levels of loneliness, and further have more improvement on subthreshold depression, when compared to participants who performed SP exergames. The results support the hypotheses on play mode and psychosocial well-beings (H2 and H4), which are developed from previous studies (Lazzaro, 2004; Singh and Misra, 2009; Zhang and Kaufman, 2016b). The significant mediation link found in the study has further deepened the research domain of exergames and literature concerning psychosocial benefits. Several previous studies have indicated that exergames led to less loneliness and depression (Rosenberg et al., 2010; Kahlbaugh et al., 2011). The current study extended the findings by emphasizing the possible influencing role of play mode, that is, MP exergames have better effects over SP ones on alleviating loneliness and subthreshold depression.

By allowing different players to interact in a team, MP exergames could foster both virtual and real social interactions among players, thereby reducing the risk of social isolation and loneliness (Mueller et al., 2003; Staiano and Calvert, 2011). In a narrative review, Brox et al. (2011) further highlighted the importance of using MP exergames among older adults. They believed that MP exergames increased social interaction between older adults, reduced their social isolation and prevented loneliness and subthreshold depression. The current study provided important evidence to support the assumption from previous research, and confirmed the strong effects of play mode on loneliness and subthreshold depression among older adults.

However, the mediating role of social support was not supported in the study, which differed from the given expectation. Although MP exergames were assumed to increase social interaction among players (Mueller et al., 2003; Staiano and Calvert, 2011), in this study they did not lead to higher social support than SP exergames. Social support generally consists of two major categories, namely emotional and instrumental support (Wills, 1985; Langford et al., 1997). In the context of exergaming, MP simply cannot provide better instrumental support (e.g., tangible support or real assistance in daily life) over the SP. The increased social bonding and communication in MP exergaming context may not guarantee the rise in emotional support in real life within only 6 weeks. The group setting in SP may be another possible explanation of the non-significant finding.

Although the initial model did not reflect the dependency of social support and loneliness, the final model supports the effects of social support on loneliness and subthreshold depression in exergames. It shows that social support not only has a direct anti-depressive effect, but also has an indirect effect via loneliness. While the significant relationship among social support, loneliness, and depression has been examined for long time in the general context (Chi and Chou, 2001; Singh and Misra, 2009), many recent studies are exploring this relationship in the adoption of new technologies (He et al., 2014; Chopik, 2016). For example, a cross-sectional study from He et al. (2014) indicated that social support was negatively associated with depression of Internet addicts whereas loneliness plays a mediating role. In consistent with these studies, the current study extends the significant relationship to the context of exergaming. Meanwhile, despite social support not being affected by play mode, its variances, which were probably affected by other external factors, assumed a significant role in the effects of play mode on subthreshold depression. These findings imply that social support may act as a moderator in the relationship between play mode and subthreshold depression. Future studies are needed to examine this assumption.

The current study does incur some limitations. Firstly, the small sample size and short intervention time may affect the generalizability of the key results, which was mainly caused by the difficulties in recruiting and managing older participants. Although no strict and clear criteria are required in the sample size of SEM studies, many researchers in the field recommend using more than 200 subjects (Kline, 2011; Tabachnick and Fidell, 2013). The findings of path analysis in this pilot study should be interpreted with caution. Secondly, the proposed model only involved two possible mediators. Future research may include more variables and draw a more complete picture on the influencing mechanism of play mode effect on subthreshold depression among older adults in exergaming. Thirdly, only Wii Tennis was used in two conditions, due to the lack of available exergames in the current market. The key conclusions should be further examined with the use of other suitable exergames in the future. Fourthly, the PHQ-9 scale has a low internal consistency at the baseline. This problem might be caused by the screening process where we only recruited participants with a limited range of PHQ-9. Therefore, the conclusions of ANOVA should be further confirmed in future studies. Lastly, it would be more worthwhile to test the interaction effect between play mode and other exergame factors in future studies, such as comparing SP and MP in both non-exercise games and exergames.

To conclude, the study compared the effects of SP and MP exergames, and supported that play mode was important in affecting the anti-depressive effects of exergames. The model examination presents a novel understanding of the mechanism inside this influencing process. Specifically, multiple-player exergames promoted better improvements on subthreshold depression and loneliness among older adults, when compared to single-player exergames. The study highlights several practical and theoretical implications applicable for both game design and mental health research. For serious game design, evidence from current research emphasizes the importance of designing multiple-player exergames for older adults with a healthcare purpose. Schutter and Abeele (2008) conducted a participatory design study and their findings suggested that older adults preferred multiple-player games when they were involved in the design process of digital games. Therefore, to maximize the psychosocial effects, exergames should be designed with a preference of multiple-player mode for older players, and involve more elements to increase social interactions and support. For mental health research, the findings also contribute to exiting literature by providing additional insight into a possible causal association lining play mode and subthreshold depression in exergames. Most of the previous studies examined the effects of play mode in the behaviors and perceptions, such as motivation, gaming performance, and experience (Smyth, 2007; Peng and Hsieh, 2012; Chen et al., 2015). The current study extended and tested its possible link to mental health improvements. It further inspires the theoretical discussion of play mode effect under a broader domain of exergames for mental health, such as anxiety and dementia. Following the discussion of multiple-player mode in exergames, it may also be interesting for the future study to explore who to play with, especially on the psychosocial effects of inter-generational exergames.

## Data Availability Statement

The raw data supporting the conclusions of this article will be made available by the authors, without undue reservation.

## Ethics Statement

The studies involving human participants were reviewed and approved by the Institutional Review Board (IRB) of Nanyang Technological University, Singapore (IRB-2014-07-039). The patients/participants provided their written informed consent to participate in this study.

## Author Contributions

JL: conceptualization, methodology, investigation, formal analysis, and writing. YT: conceptualization, supervision, project administration, resources, and reviewing and editing. SF: supervision, reviewing and editing, and funding acquisition. All authors contributed to the article and approved the submitted version.

## Conflict of Interest

The authors declare that the research was conducted in the absence of any commercial or financial relationships that could be construed as a potential conflict of interest.

## Footnotes

1 QuickCalcs: www.graphpad.com/quickcalcs/randomize1.cfm

2 www.phqscreeners.com
